# Risk analysis of colorectal cancer incidence by gene expression analysis

**DOI:** 10.7717/peerj.3003

**Published:** 2017-02-15

**Authors:** Wei-Chuan Shangkuan, Hung-Che Lin, Yu-Tien Chang, Chen-En Jian, Hueng-Chuen Fan, Kang-Hua Chen, Ya-Fang Liu, Huan-Ming Hsu, Hsiu-Ling Chou, Chung-Tay Yao, Chi-Ming Chu, Sui-Lung Su, Chi-Wen Chang

**Affiliations:** 1 National Defense Medical Center, Taipei, Taiwan; 2 Department of Otolaryngology-Head and Neck Surgery, Tri-Service General Hospital, National Defense Medical Center, Taipei, Taiwan; 3 Section of Biostatistics and Informatics, Department of Epidemiology, School of Public Health, National Defense Medical Center, Taipei, Taiwan; 4 Department of Pediatrics, Tungs’ Taichung MetroHarbor Hospital, Wuchi, Taichung, Taiwan; 5 Department of Medical Research, Tungs’ Taichung MetroHarbor Hospital, Wuchi, Taichung, Taiwan; 6 Department of Nursing, Jen-Teh Junior College of Medicine, Nursing and Management, Miaoli, Taiwan; 7 Department of Education and Research, Shin Kong Wu Ho-Su Memorial Hospital, Taipei, Taiwan; 8 Division of General Surgery, Department of Surgery, Tri-Service General Hospital Songshan Branch, National Defense Medical Center, Taipei, Taiwan; 9 Department of Nursing, Far Eastern Memorial Hospital and Oriental Institute of Technology, New Taipei City, Taiwan; 10 Department of Emergency, Cathay General Hospital and School of Medicine, Fu-Jen Catholic University, Taipei, Taiwan; 11 RN, PhD, Assistant Professor, School of Nursing, College of Medicine, Chang Gung University & Assistant Research Fellow, Division of Endocrinology, Department of Pediatrics, Linkou Chang Gung Memorial Hospital, Taiwan; 12 Department of Nursing, College of Medicine, Chang Gung University, Taoyuan, Taiwan

**Keywords:** Cancer, Microarray analysis, Gene expression, Gene ontology, Prediction analysis for microarrays

## Abstract

**Background:**

Colorectal cancer (CRC) is one of the leading cancers worldwide. Several studies have performed microarray data analyses for cancer classification and prognostic analyses. Microarray assays also enable the identification of gene signatures for molecular characterization and treatment prediction.

**Objective:**

Microarray gene expression data from the online Gene Expression Omnibus (GEO) database were used to to distinguish colorectal cancer from normal colon tissue samples.

**Methods:**

We collected microarray data from the GEO database to establish colorectal cancer microarray gene expression datasets for a combined analysis. Using the Prediction Analysis for Microarrays (PAM) method and the GSEA MSigDB resource, we analyzed the 14,698 genes that were identified through an examination of their expression values between normal and tumor tissues.

**Results:**

Ten genes (*ABCG2*,* AQP8*,* SPIB, CA7*,* CLDN8*,* SCNN1B*,* SLC30A10*,* CD177*, *PADI2*, and *TGFBI*) were found to be good indicators of the candidate genes that correlate with CRC. From these selected genes, an average of six significant genes were obtained using the PAM method, with an accuracy rate of 95%. The results demonstrate the potential of utilizing a model with the PAM method for data mining. After a detailed review of the published reports, the results confirmed that the screened candidate genes are good indicators for cancer risk analysis using the PAM method.

**Conclusions:**

Six genes were selected with 95% accuracy to effectively classify normal and colorectal cancer tissues. We hope that these results will provide the basis for new research projects in clinical practice that aim to rapidly assess colorectal cancer risk using microarray gene expression analysis.

## Introduction

Bioinformatics is a scientific field that has gained popularity worldwide. In particular, bioinformatics represents a multidisciplinary field of biology, information technology and mathematics, and harnesses the power of the Internet. Advancements in molecular biology technologies have led to the emergence of extremely large datasets, commonly known as “big data.” It is increasingly difficult to manage biological and chemical data with traditional methods because of both the larger size and increasing complexity of these datasets. Novel computing technologies and perspectives applying effective bioinformatics methods are required to accurately manage various data sources.

Colorectal cancer (CRC) is one of the leading cancers worldwide (Chu et al., 2014a; Chu et al., 2014b). Several studies have revealed that CRC screening can detect and reduce its progression toward an advanced disease stage, which leads to better overall survival. In the past, traditional CRC screening methods have included fecal blood testing, flexible colonoscopy and barium enema X-ray (Jemal et al., 2008). However, these tests are conducted in clinical practice with some limitations, such as variable sensitivity (37–80%) and potential die *t*-test interactions (Nannini et al., 2009). Therefore, new biomarkers have been developed for the detection of CRC to improve the sensitivity and specificity of detection (Chang et al., 2014a).

Microarray assays can be applied to acquire information on thousands of genes simultaneously and provide clear insights into genomic alterations related to the process of colorectal carcinogenesis, tumor growth, and metastasis. The results of microarray assays enable the identification of gene signatures for diagnosis, molecular characterization, prognostic analysis, and treatment prediction (Nannini et al., 2009).

Nevertheless, studies have revealed that the application of microarray analysis in clinical practice still faces certain challenges. First, there is a general lack of concordance between the results obtained from individual studies because of technique-related variations in sample collection and different types of platforms and methods (Cardoso et al., 2007). Second, there is a shortage of large-scale studies because of the relatively small number of available patient samples, which leads to reduced statistical power (Chan et al., 2008). Third, identifying data that would be the most informative and useful for the development of reliable clinical applications has been challenging (Nannini et al., 2009; Chang et al., 2014a; Chang et al., 2014b; Chu et al., 2014a; Chu et al., 2014b).

To overcome these challenges, one approach is to use the online Gene Expression Omnibus (GEO) database, which can help increase the sample size, heterogeneity of a sample, and statistical power. Several methods can be applied to analyze variations in gene expression between colorectal tumors and normal mucosa tissues to screen for significant cancer-related genes (Chou et al., 2013; Chu et al., 2014a; Chu et al., 2014b). In this study, we followed the Prediction Analysis for Microarrays (PAM) method to screen for significant CRC-associated genes that could be used as predictive markers for early cancer detection. Furthermore, Gene Ontology (GO) pathways and Gene Set Enrichment Analysis (GSEA) were employed to confirm the function and association of the candidate genes with the risk of CRC.

## Methods

### Microarray data sources

Microarray data were collected from the online GEO database between September 2011 and March 2014.

In this study, we searched the GEO database of the National Center for Biotechnology Information using the keywords “colon cancer,” “human [organism],” and “expression profiling by array [dataset type].”

**Figure 1:**
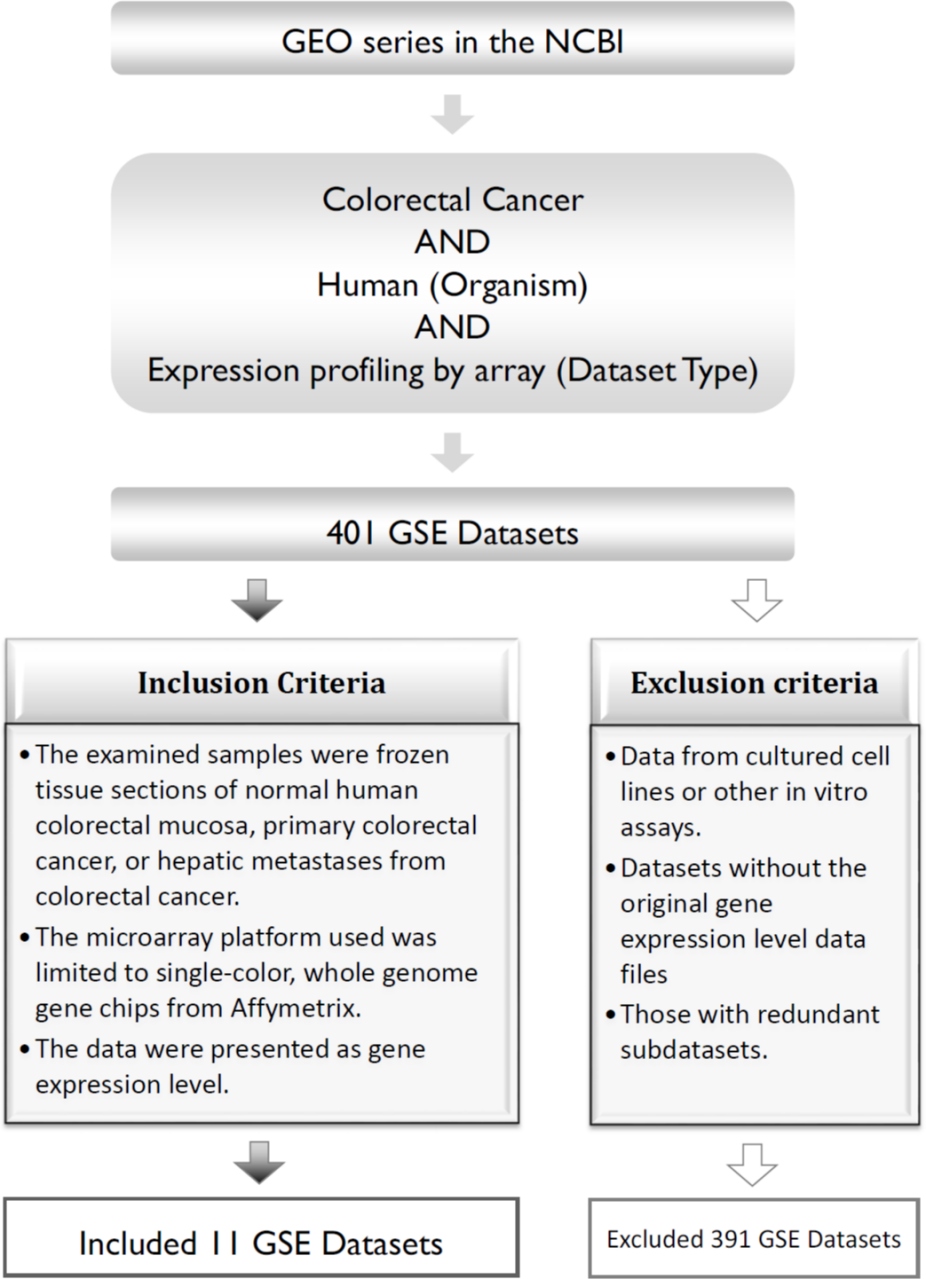
Process of pooling the 11 microarray gene expression datasets. GEO, Gene Expression Omnibus; GSE, GEO series.

The three main inclusion criteria for our data were as follows: (1) frozen tissue sections collected from primary CRC, normal human colorectal mucosa, or hepatic metastases in patients with CRC; (2) the microarray platform contained single-color, whole-genome gene chips from Affymetrix; and (3) the data were presented as the mean gene expression level. The exclusion criteria were as follows: (1) data collected from cultured cell lines or other *in vitro* assays; (2) datasets lacking the original gene expression levels; and (3) sub-datasets with redundancy (Fig. 1).

Based on these criteria, a total of 401 GEO series (GSE) datasets were excluded; therefore, 11 public microarray datasets were used for the analysis (GSE18088, GSE20916, GSE21510, GSE23878, GSE29623, GSE31595, GSE32323, GSE33113, GSE35144, GSE37892, and GSE49355), which included 717 tumor cases and 134 normal mucosa control samples (Table 1).

**Table 1:** GSE datasets included in our study.

GSE	Tissue		Total numbers	Total number of genes on chips	Type of gene chips
	Tumor ( *n* = 717 )	Normal ( *n* = 134 )			
18088	53		53	33727	HG-U133_Plus_2
20916	115	30	145	33727	HG-U133_Plus_2
21510	105	43	148	33727	HG-U133_Plus_2
23878	59		59	33727	HG-U133_Plus_2
29623	65		65	33727	HG-U133_Plus_2
31595	37		37	33727	HG-U133_Plus_2
32323	17	17	34	33727	HG-U133_Plus_2
33113	90	6	96	33727	HG-U133_Plus_2
35144	27		27	33727	HG-U133_Plus_2
37892	130		130	33727	HG-U133_Plus_2
49355	19	38	57	14713	HG-U133A

In addition, we included microarray datasets obtained from our laboratory and published by Chang et al. (2014b) (GSE4107, GSE4183, GSE8671, GSE9348, GSE10961, GSE13067, GSE13294, GSE13471, GSE14333, GSE15960, GSE17538, and GSE18105), which included 519 adenocarcinoma cases and 88 normal mucosa control cases.

### Preprocessing of microarray data

To lower the background noise of the microarray chips related the gene expression levels, data preprocessing was performed using the standard GC Robust Multi-Array Average (GCRMA) method. In addition, we also used the R language software package to conduct our study (Chu et al., 2014a; Chu et al., 2014b). This analysis of gene expression levels used the median probe expression level based on the skewed distribution of the expression levels of the probe.

Bolstad et al. (2003) proposed applying the GCRMA method over the conventional Robust Multichip Average (RMA) method. RMA analysis is performed to adjust the affinity among the nucleotides based on the different binding strengths between GC and AT provided by the Affymetrix Console. The RMA method is designed for processing Affymetrix chips. The microarrays were first preprocessed for within-study normalization using the GCRMA method, and then the calculated gene levels were estimated before the different studies were combined, while retaining only the genes that were available on all the microarrays. Next, the same preprocessing procedure was performed for between-study normalization (Chu et al., 2014a; Chu et al., 2014b).

The Affymetrix chips used in the datasets were HG-U133A and HG-U133-Plus-2, which accounted for 14,713 and 33,727 of the corresponding number of genes in our study, respectively. To obtain the expression levels of the 14,698 genes, 11 datasets were merged. Next, quantile normalization was conducted on all gene expression values (Bolstad et al., 2003).

### The PAM model

The PAM method utilizes nearest shrunken centroid methodology. The use of the PAM method may be of crucial importance for reducing not only signal noise but also the false discovery rate (FDR), which leads to the selection of the best candidate gene set (Lee et al., 2005). The PAM method is preferred because it performs better with fewer genes (Lee et al., 2005; Chu et al., 2014a; Chu et al., 2014b). Tibshirani et al. (2002) reanalyzed the leukemia microarray data of Golub et al. (1999) and confirmed 43 of the 96 genes in the microarray data using the PAM method. The results were comparable with the results of Khan et al. (2001), which were obtained using the ANN method. Furthermore, the FDR was reduced from 4 to 2 over 34 classifications.

### Functional pathway analysis

The use of pooled GEO studies was secondary because only the microarray data were available. Previous studies have proposed that testing 55 genes in any experimental model is beneficial for colon cancer biology (Chang et al., 2014a; Chang et al., 2014b; Chu et al., 2014a; Chu et al., 2014b). Therefore, in the present study, functional pathways related to the tumorigenesis of CRC were evaluated using GSEA software version 2.07. The GSEA MSigDB resource provides a collection of annotated gene sets based on different sources of information, including gene ontology, pathways, and motifs (Cardoso et al., 2007). Using the GSEA MSigDB resource, we analyzed the 14,698 genes that were identified by examining the expression values between the normal and tumor tissues.

## Results

The PAM analysis identified 10 significant candidate genes at least once after 100 repeated samplings (*ABCG2, AQP8, SPIB, CA7, CLDN8, SCNN1B, SLC30A10, CD177, PADI2* and* TGFBI)* (Table 2). Three of these genes—*ABCG2, AQP8, and SPIB*—were identified in all 100 repeated samplings. *CA7* was identified 99 times, *CLDN8* 89 times, *CNN1B* 62 times, *SLC30A10* 29 times, *CD177* five times, *PADI2* two times and *TGFBI* two times. The more frequently a gene was identified in this analysis indicates its increased importance in CRC.

**Table 2:** The centroid scores and frequency of the colorectal cancer genes in the 100 repeated samplings using the PAM method.

		CRC centroid score				NOR centroid score				Diff score (Max)
Genes	Frequency	Mean	SD	Max	Min	Mean	SD	Max	Min	
ABCG2	100	−0.023285	0.005258	−0.0121	−0.0406	0.183034	0.041345	0.3191	0.0949	0.3597
AQP8	100	−0.024511	0.005812	−0.0096	−0.0372	0.192729	0.045658	0.2925	0.0754	0.3297
SPIB	100	−0.034727	0.004733	−0.0207	−0.0456	0.273003	0.037222	0.3582	0.1625	0.4038
CA7	99	−0.051488	0.005233	−0.0429	−0.0666	0.404711	0.041172	0.5239	0.3369	0.5905
CLDN8	89	−0.010152	0.004605	−0.0015	−0.026	0.079792	0.036207	0.2044	0.0118	0.2304
SCNN1B	62	−0.004138	0.00235		−0.0098	0.032498	0.018485	0.0771	0.0002	0.0869
SLC30A10	29	−0.004566	0.002946	−0.0003	−0.0102	0.035979	0.023184	0.0804	0.0024	0.0906
CD177	5	−0.00254	0.002319	−0.0004	−0.0051	0.02006	0.018175	0.0403	0.0034	0.0454
PADI2	2	−0.00265	0.001768	−0.0014	−0.0039	0.0208	0.013859	0.0306	0.011	0.0345
TGFBI	2	0.00045	0.000354	0.0007	0.0002	−0.0033	0.002687	−0.0014	−0.0052	0.0059

**Notes.**

CRC colorectal cancer tissue NOR normal tissue

Furthermore, genes with a higher absolute centroid value were of greater importance in the CRC risk analysis because this value indicated a better ability to differentiate between cancer and normal tissues. *CA7* had the highest centroid value (0.5905), followed by *SPIB* (0.4038) and *ABCG2* (0.3597). *TGFBI* had the lowest centroid value (0.0059).

**Figure 2:**
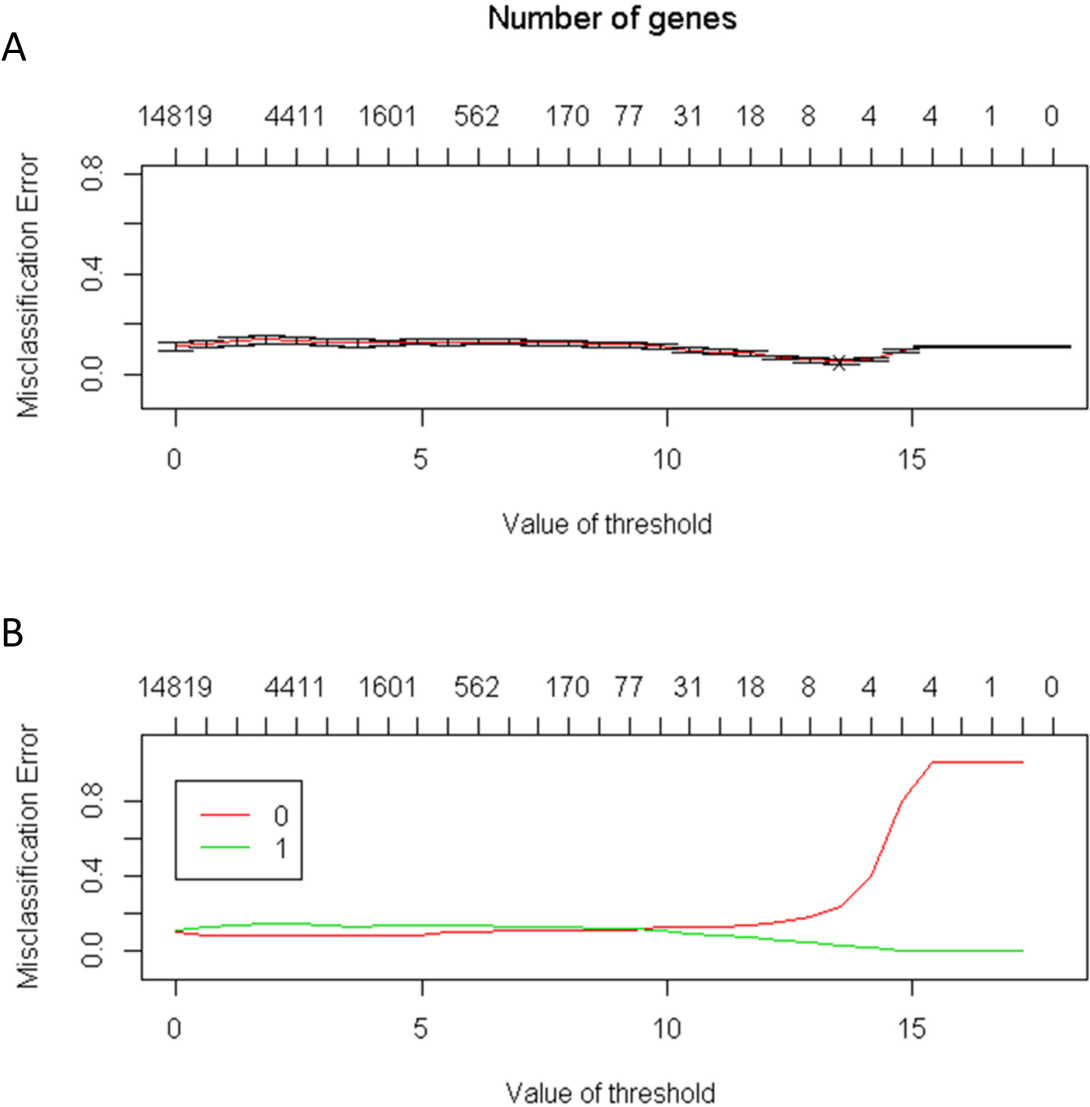
(A) The lowest threshold between the normal tissue and colorectal tumors tissue is 14; (B) The number of needed genes is between four and eight genes.

**Figure 3:**
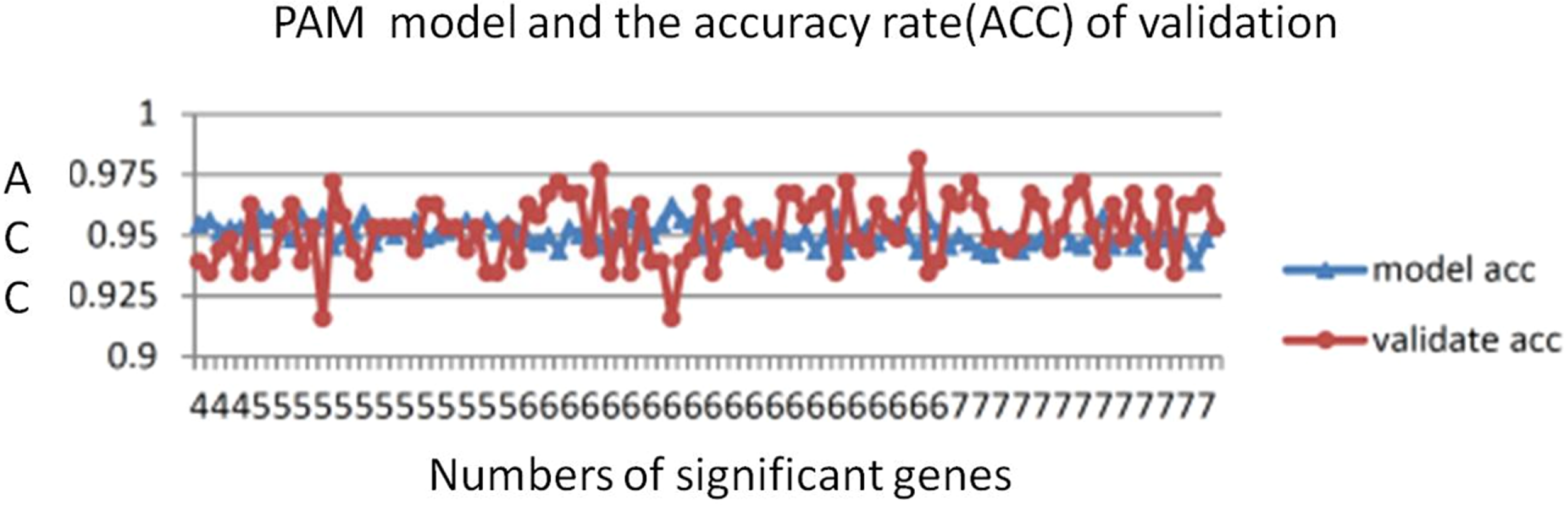
PAM model accuracy rates. The average PAM model accuracy rate was 95% (SD = 0.44). The average validation accuracy rate was 95.2% (SD = 1.33). The average number of significant genes was 5.9.

The number of genes identified using the PAM method is a good indicator of the candidate genes that correlate with CRC. The lowest threshold of the misclassification error rate to distinguish CRC from normal colon tissues was 14 of 100 repeated samples (Fig. 2). Furthermore, only six genes were required to distinguish CRC from normal colon tissues. The average accuracy rate of the model was 95% (standard deviation = 0.44), and the validation accuracy rate of the model was 95.2% (standard deviation = 1.33). The average number of significant genes obtained from the selection was six (Fig. 3)

The resulting 10 significant genes from the 100 repeated samplings were derived from Gene Ontology analysis. In the molecular function Gene Ontology category, *SLC30A10*, *ABCG2*, and *AQP8* are related to material transportation, *SPIB* and *TGFBI* are related to receptor binding, and the other genes are related to enzyme activities. In the biological process category, *SCNN1B* and *TGFBI* are specifically related to sensory perception and organ development. In the cellular component category, only *CLDN8* localizes to the plasma membrane, and the remaining genes were not annotated (Table 3).

**Table 3:** The GO terms, GO molecular function, GO biological process, GO cellular component of the 10 significant colorectal cancer genes.

Gene	GO terms	GO molecular function	GO biological process	GO cellular component
CA7	Carbonic anhydrase 7	Hydro-lyase activity	Metabolic process	
SCNN1B	Amiloride-sensitive sodium channel subunit beta	Ion channel activity	Sensory perception of taste Sensory perception of pain Cation transport Regulation of biological process	
SPIB	Transcription factor Spi-B	Sequence-specific DNA binding transcription factor activity Receptor binding	B cell mediated immunity Macrophage activation Transcription from RNA Polymerase II promoter Cell cycle Cell communication Endoderm development Mesoderm development Hemopoiesis Cellular defense response regulation of transcription from RNA polymerase II promoter	
CD177	CD177 antigen			
SLC30A10	Zinc transporter 10	Transmembrane transporter activity	Cation transport	
TGFBI	Transforming growth factor-beta-induced protein ig-h3	Receptor binding	Cell communication Cell–matrix adhesion Visual perception Sensory perception Mesoderm development Skeletal system development Muscle organ development	
PADI2	Protein-arginine deiminase type-2	Hydrolase activity	Cellular protein modification process	
ABCG2	ATP-binding cassette sub-family G member 2	ATPase activity, coupled to transmembrane movement of substances Transmembrane transporter activity	Lipid metabolic process Lipid transport	
CLDN8	Claudin-8		Cellular process	Plasma membrane Cell part
AQP8	Aquaporin-8	Transmembrane transporter activity	Transport	

## Discussion

Chang et al. (2014b) verified and compared 3 gene expression profiles for CRC using 12 GEO-online microarray databases. In addition, the authors merged three profiles and obtained a 4th profile with higher accuracy. The results of dry-lab analyses must be verified by wet-lab experiments, and conversely, the results of wet-lab experiments must be explored in dry-lab analyses. We believe that more precise experiments are needed to investigate the genes selected in the present study as in our previous publications (Chang et al., 2014a; Chang et al., 2014b; Chu et al., 2014a; Chu et al., 2014b; Ko et al., 2015; Kuan et al., 2015). The lack of dry-lab analyses may be a limitation but also provides opportunities for complete external validation in future studies. For example, we were the first to report that carbonic anhydrase VII (CA7) expression plays an initiating but not progressive role in CRC (Chu et al., 2014b) Subsequently, two studies (Kalmár et al., 2015; Yang et al., 2015) verified the role of CA7 by western blot and immunohistochemistry analyses as well as qRT-PCR analyses of clinical samples including colorectal paraffin-embedded (FFPE) tissue. Recent studies have reported that miRNAs often function as tumor suppressors or oncogenes. miRNAs related to carcinogenesis are regarded not only as diagnostic and prognostic biomarkers but also as therapeutic targets. The miR-200 family is related to TGF-*β*2 and functions in the suppression of metastasis (Kuan et al., 2015).

Furthermore, four genes, ABCG2, PADI2, CA7, and TGFBI, have been verified in previous studies as potentially correlated with colorectal cancer. Tuy et al. (2016) conducted a study of resected primary tumor specimens from 189 patients and evaluated the expression of the ABCG2 protein and drug sensitivity to SN-38 (an active metabolite of irinotecan). They also analyzed progression-free survival (PFS). Of the tumors, 60% showed higher ABCG2 expression and greater resistance to SN-38. In addition, the risk of resistance was increased by 12-fold in these tumors. PFS was lower in patients with higher expression of ABCG2. These results demonstrated that ABCG2 is a useful predictive biomarker of resistance to irinotecan and survival.

Cantariño et al. (2016) conducted a study of PADI2 expression in 98 cancer patients and 50 donors without cancer as a control group. PADI2 expression was lower or even absent in CRC. In addition, a low level of PADI2 expression in the colon mucosa was also observed in patients with ulcerative colitis. The authors concluded that a lower PADI2 expression level was associated with poorer prognosis.

Yang et al. (2015) performed real-time PCR, western blot, and immunohistochemistry analyses to evaluate the level of CA7 expression in CRC samples. Their study included two groups: a training cohort group of 228 patients to evaluate pathological features and a validation group of 151 patients from different cities in China. The authors used Kaplan–Meier and Cox proportional regression analyses to evaluate the relationships between CA7 expression and patient survival. The results showed that decreased gene expression levels of CA7 were related to disease progression. Therefore, CA7 can predict poor prognosis in patients with CRC and early-stage tumors.

Zhu et al. (2015) conducted a study of TGFBI in 115 patients using immunohistochemistry methods. Most of the TGFBI was localized in the cytoplasm in cancer tissues. High expression levels of TGFBI in the cytoplasm were related to lymph node metastasis and distant metastasis. In addition, high TGFBI was related to poor prognosis. In other words, TGFBI can be regarded as an indicator of poor prognosis in patients with CRC. Verification of our other six candidate genes in future studies is critically important.

Three genes—*ABCG2 AQP8* and *SPIB*—were identified by the PAM model to have significantly different expression levels between CRC and normal colon mucosa tissues from 100 repeated samplings. *ABCG2* is a half-transporter of the G subfamily of ATP-binding cassette transporter (ABC transporter) genes and is known to confer multidrug resistance. The mechanism of *ABCG2*-mediated multidrug resistance is related to the JNK1/c-Jun (c-Jun N-terminal kinase) signaling pathway (Xie et al., 2014). Andersen et al. (2015) revealed that the mRNA expression of *ABCG2* was decreased in cancer tissues. These findings underscore the importance of ABC transporters in the early steps of carcinogenesis and suggest that tumor formation might be related to epithelial barrier dysfunction. In addition, Kang et al. (2015) proposed that ABCG2 could be utilized as a prognostic biomarker. Their results indicated that patient survival after operation correlated with the expression of membranous ABCG2 tumors. Therefore, the detection of ABCG2 can not only identify a possible risk of colorectal cancer risk but also support survival prediction and treatment strategies.

SPIB is an ETS family transcription factor that is associated with the putative oncogene product PU.1. Furthermore, ETS transcription factors are involved in malignant transformation of cells and therefore are possible targets for cancer therapy (Ray et al., 1992; Oikawa, 2004). Takagi et al. (2016) revealed that SPIB expression is a novel indicator of poor prognosis in patients with diffuse large B-cell lymphoma and mediates apoptosis through the PI3K-AKT pathway. Furthermore, Ho et al. (2016) reported that high *SPIB* and *KI-67* mRNA expressions levels were associated with poor survival in patients with hepatocellular carcinoma. Therefore, SPIB may be related to not only carcinogenesis but also prognosis in colorectal cancer. Nevertheless, further studies are required to validate these findings.

AQP8 is a member of the aquaporin (AQP) family and facilitates water transport across the cell plasma membrane. Recent studies have revealed that AQP expression in tumors is related to cell extravasation, invasion and metastases (Yang et al., 2015). However, the clinical importance of AQP8 in colon cancer remains undetermined. Wang et al. (2012) reported two phenomena. The first is that AQP8 is not expressed in patients with colorectal carcinoma. The second is that AQP5 expression is associated with cancer stage, pathology differentiation, and lymph node metastasis. These findings suggest that decreased AQP8 expression and increased AQP5 expression might be related to oncogenesis.

CA7 is a member of the carbonic anhydrases gene family, which has been proposed to be related to the pathogenesis of human cancers. Indeed, CA7 is associated with poor prognosis and disease progression, particularly in the early stages of colon cancer. As a result, decreased expression of CA7 may be a poor prognostic indicator of CRC. In contrast, Bootorabi et al. (2011) reported that upregulation of CA7 expression was indicative of poor prognosis in patients with astrocytomas (Verkman, Hara-Chikuma & Papadopoulos, 2008).

CLDN8 is a member of the family of claudins, which play a role in tumorigenesis through alterations in cell interactions. In CRC tissues, CLDN1 and CLDN2 were upregulated. In contrast, CLDN5, 8, 15, and 23 were downregulated in CRC (Gröne et al., 2007; Bujko et al., 2015).

SLC30A10 is related to the methylation epigenotype and molecular genesis of CRC (Yagi et al., 2010). In addition, SCNN1B is associated with hypermethylation in CRC. Mitchell et al. (2014) proposed that tumorigenesis results from epigenetic changes, including hypermethylation (Kim et al., 2011). Guillaume et al. reported that SCNN1B is hypermethylated in renal cell carcinoma and is considered a new epigenetic marker for clear cell kidney carcinoma, which suggests it is a viable diagnostic test of urine or blood samples.

CD177 has been proposed as a stem cell factor receptor. Collet et al. (2015) reported that tumor stem cells likely contribute to the metastatic potential of cancers and may be responsible for chemotherapy resistance and induction of dormancy in tumors. Therefore, the detection, isolation, and characterization of tumorigenesis remain a challenge in cancer treatment strategies. In addition, Toyoda et al. (2013) proposed that CD177 regulates tumor cell adhesion and migration in gastric cancer. In particular, increased expression of CD177 in gastric cancer is a prognostic factor for survival. PADI2 is a member of the PAD family, which is commonly associated with abnormal pathological properties of inflammation (Chang et al., 2013). McElwee et al. (2012) reported that dysregulation of PADI activity is associated with several diseases, such as chronic obstructive pulmonary disease, rheumatoid arthritis and cancer. Furthermore, the authors revealed that PADI2 might play an important role in cancer progression and may be a potential biomarker for breast cancer. Transforming growth factor-beta-induced (TGFBI) has been reported to be a linker protein. Numerous human cancers exhibit high levels of *TGFBI* gene expression. Furthermore, high TGFBI protein expression is an indicator of poor prognosis in patients with CRC (Zhu et al., 2015). Turtoi et al. (2014) also reported that the expression of TGFBI is characteristic of liver metastasis in CRC.

The present study identified certain genes associated with CRC from pooled microarray datasets from several studies. Compared with previous studies, we used a similar method but found different gene expression profiles associated with CRC. Because our studies complement each other, the compatibility of the results is more impressive. These genes were found to be involved in the regulation of upstream, midstream, and downstream molecular signaling pathways, and their expression could be explained by gene collinearity because the genes were highly correlated. However, studies have reported that DNA microarray data might have collinearity problems among the gene expression data (Lee & Zee, 2008; Falgreen et al., 2015). Future studies should confirm the collinearity of these genes.

In a large study of cancer, Andrew et al. analyzed the gene expression signatures of approximately 18,000 human tumors across 39 malignancies. However, our study was more specific for colorectal cancer and provided a detailed examination of survival and carcinogenesis of one cancer type. The prior study provided a wide screening of all types of cancers, whereas the latter is more specific and concentrated on the genes associated with colorectal cancer (Gentles et al., 2015).

Future studies should clarify the reliability of the gene signatures observed in this study for predicting CRC risk. Furthermore, the characteristics of the candidate genes identified in this study merit further investigation using molecular biology methods, such as those involving epigenetics and genetics, DNA methylation, mRNA expression levels, mRNA interactions, and associated biochemical pathways.

Our method of investigation is not without limitations. The first limitation is that the datasets were collected from several research studies; this approach has the benefit of an increased sample size but may increase the heterogeneity due to the different types of research designs. The second limitation is that we did not identify discrepancies in CRC-related variables or variables influencing the survival of patients with CRC among the different studies. Our analysis of pooled microarray studies published in recent years revealed that several international teams have proposed different CRC gene expression profiles covering diverse candidate and verified genes, with less than 25% similarity, despite intra-observational analyses performed using various bioinformatics techniques. These discrepancies may be attributable in part to sampling variations that are not eliminated by bootstrapping, but statistical collinearity within the same pathway or associated network of gene-gene interactions is likely a more important factor and requires further study.

## Conclusions

Using the appropriate bioinformatics tools and the PAM method to obtain 100 repeated samplings, we identified 10 candidate genes that are significantly associated with CRC (*ABCG2 AQP8*, *SPIB*, *CA7*, *CLDN8*, *SCNN1B*, *SLC30A10*, *CD177, PADI2* and *TGFBI)*. On average, six genes were selected by the PAM model to effectively classify normal and CRC tissues, and the average accuracy rate was 95%. We hope that these results will provide the basis for new research projects in clinical practice to rapidly assess colorectal cancer risk using microarray gene expression analysis.

## Additional Information and Declarations

### Microarray Data Deposition

The following information was supplied regarding the deposition of microarray data:
GSE18088, Clinical outcome of stage UICC II colon cancer patients;
GSE20916, Modeling oncogenic signaling in colon tumors by multidirectional analyses of microarray data;
GSE21510, Clinical Significance of Osteoprotegerin Expression in Human Colorectal Cancer;
GSE23878, Genome Wide Expression Analysis of Middle Eastern Colorectal Cancer Reveals FOXM1 as a Novel Target for Cancer Therapy;
GSE29623, mRNA and microRNA profile in colon cancer;
GSE31595, Gene Expression Profiles in Stage II and III Colon Cancer. Application of a 128-gene signature;
GSE32323, Colorectal cancer tumors;
GSE33113, AMC colon cancer AJCCII;
GSE35144, Molecular Evaluation of Patient-Derived Colorectal Cancer Explants as a Pre-clinical Mouse Model of Colorectal Cancer;
GSE37892, A seven-gene signature aggregates a subgroup of stage II colon cancers with stage III;
GSE49355, Specific extracellular matrix remodeling signature of colon hepatic metastases.

### Data Availability

The following information was supplied regarding data availability: The raw data or code is included in the manuscript.
